# Fostering Safety Communication among Construction Workers: Role of Safety Climate and Crew-Level Cohesion

**DOI:** 10.3390/ijerph16010071

**Published:** 2018-12-28

**Authors:** Bhavana Pandit, Alex Albert, Yashwardhan Patil, Ahmed Jalil Al-Bayati

**Affiliations:** 1 Department of Civil, Construction, and Environmental Engineering, North Carolina State University, 2501 Stinson Dr., Raleigh, NC 27607, USA; bkpandit@ncsu.edu (B.P.); yspatil@ncsu.edu (Y.P.); 2 Department of Construction Management, Western Carolina University, 389 Centennial Drive, Cullowhee, NC 28723, USA; ajalbayati@email.wcu.edu

**Keywords:** construction safety, safety communication, cohesion, safety climate, occupational safety, health and safety

## Abstract

Safety communication among construction workers is fundamental to effective safety management. However, evidence suggests that poor safety communication is a common problem in construction workplaces. In fact, previous research has unveiled a number of systemic barriers to effective safety communication in the construction industry. When workers do not sufficiently communicate relevant safety hazards and appropriate injury prevention measures, unexpected injuries can follow. Therefore, research examining factors that promote or impede effective safety communication is necessary. Towards achieving this goal, the purpose of the current research was to evaluate the effect of safety climate and crew cohesion on the demonstrated safety communication levels. The goal was achieved by gathering empirical data from 57 construction workplaces in the United States. More specifically, the participating construction workplaces were visited, and data pertaining to the safety climate and crew-level cohesion were first collected using questionnaire surveys. Next, a safety communication survey instrument was administered, and the data necessary to compute network density—a social network metric that is indicative of safety communication levels was gathered. The analysis of the data suggested that a positive relationship exists between safety climate and safety communication levels. Likewise, construction crews that demonstrated higher levels of cohesion exhibited superior safety communication levels. Finally, evidence also suggested that a synergetic effect exists between safety climate and crew cohesion in improving safety communication levels.

## 1. Introduction

Construction workplaces have consistently reported an unacceptable number of injuries. For example, in the United States, construction workplaces report over 900 fatal incidents every year [1]. Likewise, the United States construction workforce experiences more than 200,000 non-fatal injuries each year [2]. Similar patterns have been reported from most nations including the United Kingdom, Canada, Australia, Hong Kong, and others [3,4,5,6]. These incidents result in substantial costs, lost productivity, and unnecessary distress to workers and their families [7,8,9]. Accordingly, much research has focused on identifying practices that can reduce the likelihood of workplace safety incidents.

Among others efforts, previous research has highlighted the importance of worker engagement in occupational safety and health management initiatives [10,11]. For example, when workers actively engage in communicating safety hazards and collaboratively identify suitable injury prevention measures, superior safety performance can be achieved [12,13]. Conversely, when safety hazards are not effectively communicated, and responsive safety measures are not identified, risky behaviors and the likelihood of injuries can increase [13,14]. Unfortunately, previous research has demonstrated that poor safety communication is a widespread issue in the constitution industry [12,13,14,15,16]. 

While the importance of effective safety communication is well understood, workplace characteristics that promote and impede effective safety communication—particularly among construction workers—has received little attention in the broader construction safety literature. Such an understanding can empower construction managers, safety professionals, and construction supervisors with actionable knowledge that can be strategically leveraged to foster better safety communication. Such efforts from the site-leadership can dramatically improve the flow of safety information and how safety is managed in workplaces.

Towards achieving this goal, the present research focused on understanding the effect of safety climate and crew-level cohesion on safety communication levels. More specifically, the goal was to assess if the safety climate levels maintained in the workplace and the cohesion-levels demonstrated by the crewmembers are useful in explaining the variability in safety communication levels.

## 2. Background

To provide the necessary foundation for the current research, the following sections discuss previous research in the area of safety communication, safety climate, and cohesion briefly. In addition, the importance of the presented research is also discussed. 

### 2.1. Safety Communication

The importance of effective safety communication in high-risk work environments is discussed in a large body of research [12,13,14,15]. For example, previous research has discussed the value of safety interactions between workers and their supervisors [17,18,19]. Likewise, research has also discussed the benefits of safety communication among workers themselves [12,13]. Much of this research has demonstrated that effective safety communication promotes the sharing of relevant safety information—which translate into desirable safety behaviors such as superior hazard recognition (e.g., identifying hazards such as trip potential, exposure to energized equipment, etc.), safety compliance (e.g., use of personal protective equipment as required by safety regulations), and safety participation (taking voluntary initiatives such as discussing safety challenges with the supervisor) [12,20,21,22]. Others have found a negative and statistically significant relationship between the effective exchange of safety information (e.g., safety hazards, safety management practices) among workers and workplace injury rates [13,21].

Based on such evidence, a large number of employers encourage their workers to participate in safety communication efforts by implementing toolbox talks and pre-task safety planning sessions [23,24]. However, desirable levels of communication are often not achieved among workers in most construction workplaces [12,13,14,15]. For example, Borys [25] found that most workers are largely unengaged when safety hazards and injury prevention methods are identified and discussed during safety planning sessions. More specifically, Borys [25] found that only one crewmember or the foreman generally completed the necessary safety planning paperwork (e.g., job safety analysis report), while the other workers remained largely unengaged. Unfortunately, when safety hazards are not sufficiently communicated, the likelihood of hazard exposure and injuries increase [12,26].

A few efforts have unveiled systemic barriers to effective safety communication in construction workplaces. For example, differences in culture, gender, and language proficiency have been acknowledged as important impediments to effective safety communication in previous research [13,14,15,16,27]. For example, previous efforts have found structural barriers to the flow of safety information to female crewmembers in mixed-gender crews [14]. Others have found that, the lack of interest in safety issues, the macho culture that is characteristic of male-dominated industries, workplace productivity pressures, and insufficient knowledge on safety hazards among workers can hamper effective safety communication [12,28,29].

While the issue of poor safety communication is discussed in the literature, approaches and intervention that employers and supervisors can adopt to foster better safety communication among workers has received little attention. Because effective safety communication can yield substantial benefits, the present research focuses on evaluating workplace characteristics that promote the open and frequent exchange of safety information.

### 2.2. Safety Climate

Safety climate measures are indicative of the priority and value assigned to workplace safety in a given workplace or within a particular crew [30]. These measures are generally captured using a variety of factors that include management support, worker involvement, and project-level safety practices and characteristics—which are all known to be necessary for effective safety management [31,32]. Accordingly, past research has established safety climate as one of the most robust predictors of safety performance [5,30].

Past research also discusses the benefits of establishing a positive safety climate. For example, Christian et al. [33] demonstrated that workers representing workplaces with a more positive safety climate are more likely to comply with safety rules and regulations. Similarly, Clarke [34] found evidence that workers are more likely to participate enthusiastically in safety-focused efforts such as safety training when a more positive safety climate is maintained. Others have found evidence of a relationship between safety climate and superior hazard recognition and the adoption of effective injury prevention methods [35,36]. In contrast, workplaces with poor safety climate levels generally report a large number of injuries and are often associated with higher underreporting rates [5,21,33,37,38].

While much research has focused on understanding the benefits of cultivating a positive safety climate, its role in fostering the exchange of safety information among workers remains unexplored. Such knowledge will be useful given the number of benefits associated with superior safety communication levels as discussed earlier.

### 2.3. Crew-Level Cohesion

Cohesion among workers and employees in the context of teams has been investigated in a number of previous efforts [39,40]. Much of this research, similar to the current effort, has defined cohesion among team/crew members as the degree or measure of bonding among members of the team or the crew that can result from a number of underlying reasons [41]. For example, when team members collaboratively pursue and value a common goal, they are likely to exhibit higher levels of cohesion [42]. Team members are also likely to exhibit higher levels of cohesion when they experience a sense of belonging or connectedness due to the nature and social features of the team (e.g., cooperation, social support, etc.) [41,42].

Apart from research that focuses on examining factors that promote cohesiveness among team members, research has also focused on evaluating the benefits and the downsides of cohesion among team members [41,43,44]. For example, previous research had demonstrated that higher cohesion levels can translate to higher level of cooperation, information sharing, and satisfaction among team members [42,45]. Others have demonstrated that cohesion levels are often associated with higher productivity rates and higher quality of services and outcomes [42,46,47]. Although potential downsides to cohesiveness such as groupthink where differing perspectives are censored by team members has been found [48], much of the research has highlighted the positive effects and benefits of cohesion in the context of teams [42].

More specific to the construction industry, higher levels of cohesion at the project level has been linked with better cost control, superior quality of the built environment, and lower turnover rates among project personnel [49]. Evidence has also suggested that higher levels of cohesion are associated with superior crew-level performance in areas including safety, productivity, and schedule performance [50].

While there is preliminary evidence suggesting that crew-level cohesion can be associated with superior safety performance based on a composite score which includes injury rates and the number of near misses [50], the mechanism through which this is achieved remains unclear. To advance knowledge in this area, the current research seeks to examine if higher levels of cohesion can foster higher levels of safety communication.

## 3. Research Objectives and Contributions

As discussed above, while the benefits of safety communication are known and discussed in previous research, there is a dearth of research on how superior safety communication can be achieved among construction workers in a crew. Accordingly, the objective of the presented research was to examine workplace factors and characteristics that foster superior safety communication levels. Towards achieving this objective, the current study examined the role of safety climate and crew-level cohesion on safety communication levels.

Because workplaces and crews that value and prioritize safety demonstrate higher levels of safety climate [30,37] and desirable safety behaviors, we hypothesized that such construction crews will also exhibit higher levels of safety communication. In other words, it was expected that when construction workplaces and crews prioritize safety (i.e., higher safety climate), they are more likely to exchange safety information more effectively at the workplace. Accordingly, the following hypothesis was tested:

**Hypothesis** **1.** *Safety climate will be positively associated with safety communication levels such that construction crews that demonstrate higher levels of safety climate will exhibit higher levels of safety communication.*

As discussed above, evidence from previous research also suggests that cohesion promotes cooperation and information sharing [42,45]. However, the role of cohesion in exchanging safety information, in particular, has not been examined in the construction context. Based on previous evidence that cohesion facilitates the exchange of information among individuals or groups of individuals [42], it was expected that crews that were more cohesive will share safety information more effectively and freely. Accordingly, the following hypothesis was tested.

**Hypothesis** **2.** *Cohesion will be positively associated with safety communication levels such that construction crews that demonstrate higher levels of cohesion will exhibit higher levels of safety communication.*

After evaluating the effect of safety climate and crew-level cohesion on safety communication levels independently, the research also focused on evaluating if there were synergetic benefits (i.e., interaction effects) of maintaining superior safety climate and crew-level cohesion. In other words, the objective was to assess if cultivating a more positive safety climate and promoting crew-level cohesion can yield even higher levels of benefits (i.e., safety communication levels) that go above and beyond (i.e., interaction effects) their independent effects examined as part of Hypothesis 1 and Hypothesis 2. Accordingly, the following hypothesis was tested.

**Hypothesis** **3.** *The synergy between safety climate and crew-level cohesion will yield higher levels of safety communication among construction workers.*

The presented research focuses on examining workplace factors and crew-level characteristics that can potentially affect safety communication levels among construction workers at the crew-level. The effort is necessary given that poor safety communication is widespread in the construction industry.

## 4. Research Methods

To test the proposed hypotheses, the research team embarked on an effort to gather empirical data from a convenience sample of construction projects. As part of this effort, data relevant to the hypotheses were gathered from 57 projects that are located in the Southeastern United States that expressed their willingness to participate in the effort. The participating projects included commercial projects such as shopping centers and business offices (36.8%), infrastructure projects such as highway construction and maintenance (19.3%), industrial projects that included manufacturing facilities and warehouses (15.8%), residential projects such as apartment complexes and single-family homes (14%) and miscellaneous project types such as educational and university facilities. Each of the participating projects were at least 15% complete at the time of the visit and data collection effort.

From each of the participating projects, a crew that included at least four crew members were recruited with the assistance of the site personnel. Only one crew was recruited from each project to ensure the variability in safety climate and other project-specific characteristics. To qualify as a crew, it was necessary that the crew-members planned and worked on a single task collectively and collaboratively on a regular basis and self-identified as a crew. It was also necessary that all of the crew members of a particular crew were available and willing to participate in the study to ensure the collection of reliable and complete crew-level data. Likewise, to ensure the collection of reliable safety communication, safety climate, and cohesion data, it was required that the crew members worked together for at least the three prior months. Most of the crews focused on a particular trade (81%) that included civil, mechanical and plumbing, electrical, carpentry, and site preparation work. The remaining crews included workers representing two or more trades that worked in unison as a single crew.

In each of the participating projects, the data collection effort focused on gathering the safety climate, the cohesion, and the safety communication data. The following sections describes the data collection and data handling approach that was undertaken as part of the research effort.

### 4.1. Collection and Handing of Safety Climate Data

To ensure the collection of reliable data, a safety climate scale designed and validated for the construction industry, in particular, as part of a National Institute of Occupational Safety and Health (NIOSH) research effort was adopted [51]. The safety climate measurement scale included 19-items that were categorized under four dimensions that included commitment from the management, support from the supervisor or foreman, safety practices and policies adopted at the workplace, and work-related pressure.

The scale included 7 items under the *commitment from the management* dimension including statements such as *“the site management thinks that job-site safety is more important than job schedules and deadlines”* and *“the site safety personnel steps-in to stop unsafe operations whenever that occurs.”* The support from the supervisor or foreman dimension included six items such as *“our foreman/supervisor makes sure we follow site safety rules and procedures very closely.”* Likewise, the safety practices and policies dimension included 4 statements such as *“toolbox talks about safety are given regularly,”* and the workplace pressure dimension included 2 items that included *“sometimes we (i.e., workers) ignore a safety rule or policy in order to carry out an assignment to meet the schedule.”*

The safety climate scale was administered to each of the workers in the participating crews and they were asked to report their level of agreement with the statements using a 7-point Likert scale (1 = strongly disagree, 2 = disagree, 3 = somewhat disagree, 4 = neither agree nor disagree, 5 = somewhat agree, 6 = agree, 7 = strongly agree). After the data was gathered, two negatively worded items in the survey were reverse coded prior to calculating the aggregate safety climate score for each crew (the remaining items were positively worded).

To calculate the crew-level safety climate score, the average score across the 19-items were first computed for each worker in a crew. Subsequently, the average of the scores across each of the workers (i.e., average of averages) representing a crew was computed as the crew-level safety climate score. Accordingly, this resulted in 57 safety climate scores representing each of the participating construction crews.

### 4.2. Collection and Handling of Cohesion Data

Because a scale to measure cohesion levels particularly among construction crew members does not exist, the first step prior to data collection was to identify a list of items that are indicative of cohesion. The items were identified through a review of literature where cohesion was measured in the context of teams and groups. A number of measurement scale items (~30) were identified from a number of sources that measured cohesion in the context of education, organizations, sports, and the armed forces (e.g., military) [43,52,53,54,55,56,57]. The examined measurement scales have also been used in other high-stake team or group context including among fire-fighters and healthcare professionals [58,59]. The items from these sources were cataloged and were reviewed by a panel of 4 construction safety professionals with a cumulative experience of over 107 years in the construction industry. The objective of the review was to identify scale items that were relevant to the construction crew context.

After eliminating items that were irrelevant to the construction context and choosing selective items when there was substantial overlap between the items, the expert panel recommended 12 items that can be used to measure cohesion levels among construction crew members. The expert panel also recommended few modifications to the items to better fit the construction crew context and suggested that each of the items be weighted equally similar to the previous studies from where the items were adopted. The 12 items in the final form that were adopted to measure cohesion are presented in Table 1.

Similar to the safety climate survey, the crew-cohesion measurement scale (i.e., see Table 1) was presented to each of the workers in each of the participating crews. For each item, the workers provided their level of agreement using a 7-point Likert scale (1 = strongly disagree, 2 = disagree, 3 = somewhat disagree, 4 = neither agree nor disagree, 5 = somewhat agree, 6 = agree, 7 = strongly agree).

Like the safety climate scores, the crew-level cohesion was computed in two steps. First, the average response across the 12 items was calculated for each worker to represent the cohesiveness score experienced by the individual workers. Next, the average of the scores across the workers representing each of the crews were computed. Accordingly, this resulted in 57 crew-level cohesion scores representing each of the participating construction crews.

### 4.3. Collection and Handling of Safety Communication Data

**Figure 1:**
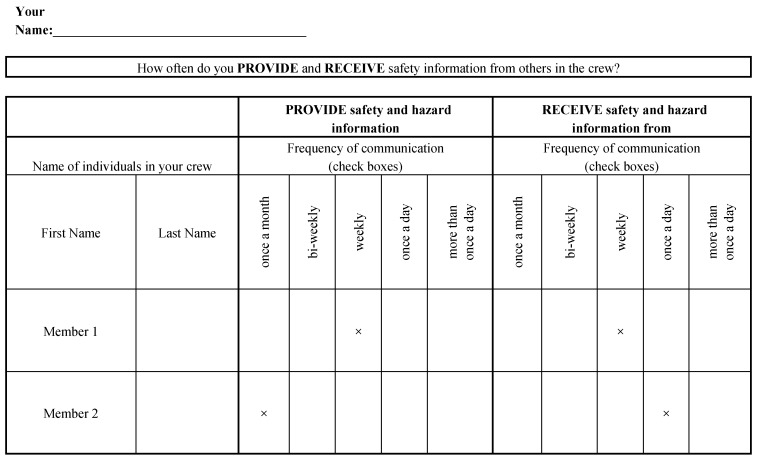
Survey instrument to gather safety communication data.

Safety communication data for each participating crew was gathered using a survey instrument of the format shown in Figure 1 [13]. As can be seen, the leftmost columns included a list of the crew members in a particular crew. This list was pre-populated prior to the administration of the survey instrument to the participating crews. It is important to note that Figure 1 only presents two member rows for illustrative purposes. However, the administered survey instrument included sufficient rows to include information that was relevant to all members in a crew (i.e., all other crew members that a particular respondent can interact with).

A copy of the survey instrument was administered to each of the crew members in a crew. Each of the crew members were asked to indicate (1) the frequency with which they provide safety information to each of the remaining crew members and (2) the frequency with which they receive safety information from each of the remaining crew members. As an illustration, the survey instrument presented as Figure 1 includes example responses from a respondent in a crew. As shown, the responded indicated that he/she provide safety information to a crew member named *Member 1* on a weekly basis. Likewise, the respondent receives safety information from *Member 1* on a weekly basis. On the other hand, the respondent provides safety information to *Member 2* on a monthly basis, but receives safety information from *Member 2* only on a weekly basis.

Using the information obtained from each of the crew members in a crew, a social network metric known as the network density, was computed for each participating crew [60]. The network density metric captures the level of communication or how well members in a crew or group are connected.

**Figure 2:**
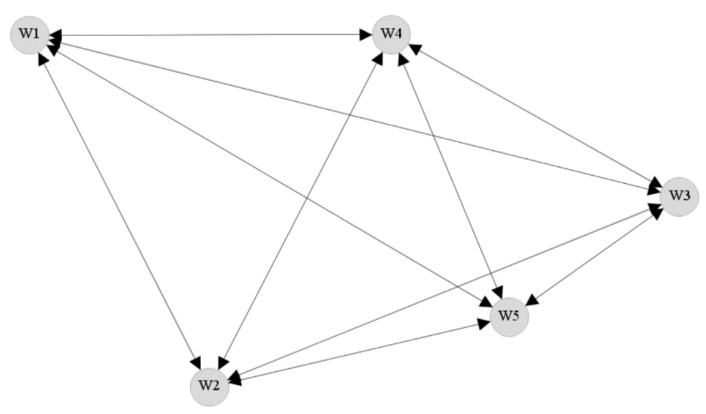
Example complete unweighted social network graph.

Two forms of network densities are commonly discussed in the literature—namely unweighted network density and the weighted network density [60,61]. The unweighted network density is usually calculated for networks where each of the connections are given the same weightage or importance. An example unweighted network is presented as Figure 2. Because each of the connections are given the same importance and each of the members (e.g., W1) in the network are connected to every other member, the network is called a complete network, and the corresponding unweighted network density is equal to 1. Alternatively, if none of the members in the network were connected (i.e., no flow of information), then the corresponding unweighted network density will be equal to 0. The unweighted network density can be more formally calculated using the expression presented in Equation (1).

(1) $$Unweighted\;Network\;Density=\frac{C}{n\;\left(n-1\right)}$$

where, C is the number of ties or connections present in the network or crew; n is the number of members or workers in a particular network or crew, and n (n−1) is the maximum number of ties or connections theoretically possible in the network that includes n number of members or workers.

When weights are assigned to the connections in a network, the weighted network density can be calculated using Equation (2). The weights are generally used to capture the frequency or the strength of the connections in a network.

(2) $$Weighted\;Network\;Density=\frac{\sum W}{n\;\left(n-1\right)}$$

where, W is the weight of a tie or connection in the network between particular crew members; n is the number of workers in a particular network or crew, and n (n−1) is the maximum number of ties or connections theoretically possible in the network that includes n number of workers.

Because the frequency with which safety information was exchanged was gathered in the current study, the frequency was used as the weights. For example, if a worker provided safety information to another worker once every day, the weight that was assigned to the connection was 0.125 (i.e., 1 interaction/8-hour work-period = 0.125). Using the same approach, the weights assigned to each of the other frequencies were as follows: more than once a day = 0.25 (i.e., 2 interaction/8-hour work-period = 0.25), once a week = 0.025 (i.e., 1 interaction/40-hour work-period = 0.025), once a two-weeks = 0.0125 (i.e., 1 interaction/80-hour work-period = 0.0125), and once a month = 0.00625 (i.e., 1 interaction/160-hour work-period = 0.00625). Accordingly, using Equation (2), a unique weighted network density score was calculated for each of the participating crews (i.e., 57 nos.).

It is important to note that the survey instrument as presented in Figure 1 captured the same information from the perspective of two workers. For example, member 1 could report that he/she provides safety information to member 2 on a daily basis; whereas member 2 may report that he/she only receives safety information on a weekly basis from member 1. When such disagreements existed the lesser of the two reported frequencies was used conservatively. Accordingly, for the example scenario provided above, the computation assumed that member 1 provides safety information to member 2 on a weekly basis.

## 5. Data Analysis and Results

As discussed above, the gathered data resulted in a unique safety climate score, a unique crew-level cohesion score, and a unique weighted network density score (henceforth referred to as the safety communication score) for each of the 57 participating projects. Using this data, descriptive analysis was first performed, and then the proposed hypotheses were tested.

Table 2 presents the means, standard deviations, and the zero-order correlations between the study variables. As can be seen, the mean safety climate score of the participating workplaces was 5.878 which lies between somewhat agree and agree in the Likert scale that was adopted in the current study effort. Likewise, the mean crew-level cohesion score (i.e., 5.986) was slightly less than agree in the adopted Likert scale. The mean safety communication score was 0.146 which suggests the participating crews, on average, achieved roughly 58% of the maximum theoretical value of 0.25 (i.e., 0.146/0.25)—which is indicative or a crew where each crewmember provides and receives safety information more than once a day.

### 5.1. Role of Safety Climate in Fostering Safety Communication (Hypothesis 1)

Hypothesis 1 predicted that safety climate scores will be positively associated with safety communication scores—suggesting that a more positive safety climate will foster higher levels of safety communication. To test the hypothesis the linear regression model presented as Equation (3) was estimated. As can be seen, the safety communication score was modeled as the dependent variable, and the safety climate score was modeled as the independent variable. The results of the hypothesis testing are presented in Table 3.

(3) $$SCL_{crew}=i_{1}+aSC_{crew}+\varepsilon_{1}$$

where, $SCL_{crew}$ represents the safety communication levels in the participating crews, $SC_{crew}$ represents the demonstrated safety climate scores of the participating crews, $i_{1}$ represents the intercept of the regression model, and $\varepsilon_{1}$ is the error term associated with the estimation of the safety communication levels using the safety climate scores.

As can be seen, the results suggest that for every unit increase in the safety climate levels, the safety communication levels increased by 0.026 units. In addition, the relationship was found to be statistically significant. Accordingly, the prediction that safety climate will foster superior safety communication is supported. In addition, the results suggest that safety climate was able to explain 9.2% of the variability in the safety communication levels.

### 5.2. Role of Crew-Level Cohesion in Fostering Safety Communication (Hypothesis 2)

Like hypothesis 1, hypothesis 2 predicted that the crew-level cohesion will be positively associated with the safety communication levels—suggesting that the crew-level cohesion can foster safety communication. To test the hypothesis, the linear regression model presented as Equation (4) was estimated where the safety communication score was modeled as the dependent variable and the crew-level cohesion scores were modeled as the independent variable. The results of the hypothesis testing are presented in Table 4.

(4) $$SCL_{crew}=i_{2}+bC_{crew}+\varepsilon_{2}$$

where, $SCL_{crew}$ represents the safety communication levels in the participating crews, $C_{crew}$ represents the demonstrated crew-level cohesion scores of the participating crews, $i_{2}$ represents the intercept of the regression model, and $\varepsilon_{2}$ is the error term associated with the estimation of the safety communication levels using the crew-level cohesion scores.

As shown in Table 4, the results suggest that for every unit increase in the crew-level cohesion score, the safety communication levels increased by 0.031 units. In addition, like the relationship between safety climate and safety communication levels, the relationship between crew-level cohesion and safety communication levels was also found to be statistically significant. The results also suggested that the crew-level cohesion explained 12.1% of the variability in safety communication. Therefore, the crew-level cohesion explained the variability in the safety communication levels by roughly an additional 3% over that explained by the safety climate levels.

### 5.3. Role of the Synergy between Safety Climate and Crew-Level Cohesion on Safety Communication (Hypothesis 3)

Hypothesis 3 predicted that the synergy between safety climate and the crew-level cohesion will foster even higher levels of safety communication. To test the hypothesis, the safety climate, the crew-level cohesion, and the interaction effect were regressed on the safety communication levels as shown in Equation (5). It is important to note that the coefficient e in Equation (5) captures the interaction or synergistic effect between safety climate and crew-level cohesion. Therefore, if the coefficient e is positive and significantly different from zero, then the conclusion that a synergetic effect exists can be made.

(5) $$SCL_{crew}=i_{3}+cSC_{crew}+dC_{crew}+e\;\left(SC_{crew}\cdot C_{crew}\right)+\varepsilon_{3}$$

where, $SCL_{crew}$ represents the safety communication levels in the participating crews, $SC_{crew}$ represents the demonstrated safety climate scores of the participating crews, $C_{crew}$ represents the demonstrated crew-level cohesion scores of the participating crews, $\left(SC_{crew}\cdot C_{crew}\right)$ represents the interaction between safety climate and crew-level cohesion, $i_{3}$ represents the intercept of the regression model, and $\varepsilon_{3}$ is the error term associated with the estimation of the safety communication levels in the regression model.

The results of the estimated regression model is presented in Table 5. The results indicate that a significant synergistic effect exists between safety climate and the crew-level cohesion (i.e., e = 0.042; *p*-value < 0.05; r^2^ = 0.244). In other words, the interaction between safety climate and crew-level cohesion can foster even higher levels of safety communication among workers. Alternatively, the results can be interpreted as: the relationship between safety climate and safety communication is strengthened when crews demonstrate higher levels of cohesion. The regression model that includes the synergetic effect explained 24.4% of the variability in the safety communication levels.

**Figure 3:**
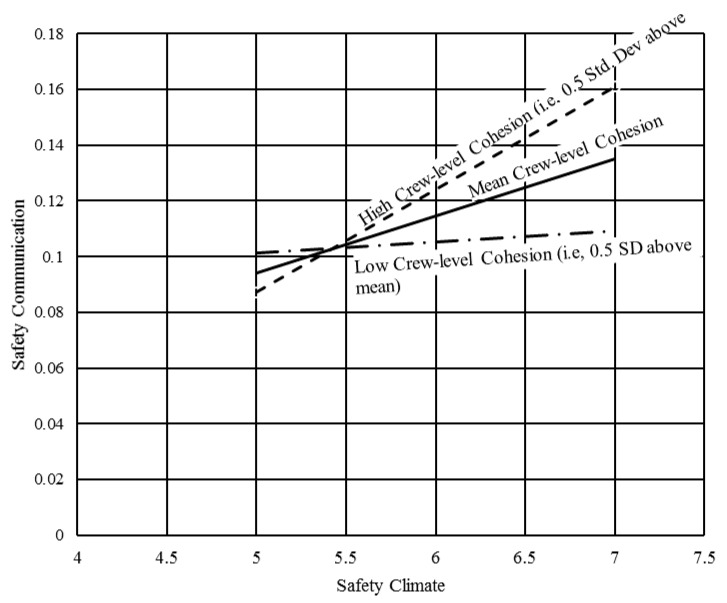
Plot demonstrating synergy between safety climate and cohesion.

To better visualize the synergistic effect, the interaction plot presented in Figure 3 was constructed using the results shown in Table 5. The solid line captures the relationship between safety climate and safety communication levels when the demonstrated crew-level cohesion is equivalent to the mean (i.e., $C_{crew}$ = 5.986 as shown in Table 2). The other two dotted lines represent the relationship between safety climate and safety communication levels when the crew-level cohesion is 0.5 standard deviations above (i.e., $C_{crew}$ = 5.592) and below (i.e., $C_{crew}$ = 6.377) the mean.

While the above-presented results demonstrate that the strength of the relationship between safety climate and safety communication increases as the crew-level cohesion increases, the results do not provide details on when the effect of cohesion is trivial versus non-trivial (i.e., significant). For example, if the crew-level cohesion is substantially low, it may not significantly affect the relationship between safety climate and safety communication.

To distinguish the range of values for which the crew-level cohesion has a non-trivial (i.e., significant) effect, the Johnson Neyman technique was adopted [62]. The process first involves estimating the conditional effect of safety climate on safety communication levels as shown in Equation (6). This equation captures the relationship between safety communication and crew-level cohesion when safety climate varies by one unit. This ratio between this relationship and the associated standard error follows a *t*-distribution and can be expressed as Equation (7). This ratio can be solved to obtain the critical value of *t*—which demarcates the value at which the crew-level cohesion transitions from being trivial to non-trivial.

(6) $$\theta_{SC_{crew}\to SCL_{crew}}=c+eC_{crew}$$

(7) $$t_{crit}=\frac{\theta_{SC_{crew}\to SCL_{crew}}}{\sqrt{s_{c}^{2}+2\left(C_{crew}\right)\;COV_{ce}+{C_{crew}}^{2}\;s_{e}^{2}}}$$

where, $\theta_{SC_{crew}\to SCL_{crew}}$ is the conditional effect of safety climate on safety communication levels for different levels of crew cohesion, $C_{crew}$ is the crew-level cohesion of the participating crews, $t_{crit}$ is the critical value of *t* when the conditional effect represented in Equation (6) becomes significant, $s_{c}^{2}$ is the squared standard error of *c*, $s_{e}^{2}$ is the squared standard error of *e*, $COV_{ce}$ is the covariance of *c* and *e*.

The computations to calculate the $t_{crit}$ was accomplished using the macro developed by Hayes and Matthes [62]. Apart from providing the $t_{crit}$, the macro also presents a continuum of conditional effects along with the results of the significance tests. The results of the test are presented in Table 6.

As can be seen, the results in Table 6 suggests that the conditional effect of safety climate on safety communication is significant only when the crew-level cohesion exceeds 6.25. Therefore, this suggests that the synergistic benefit of the crew-level cohesion and safety climate is only achieved when cohesion levels are substantially high. More specifically, the cohesion levels will need to range between agree and strongly agree, on the Likert scale that was used to capture cohesion in the current study (1 = strongly disagree, 2 = disagree, 3 = somewhat disagree, 4 = neither agree nor disagree, 5 = somewhat agree, 6 = agree, 7 = strongly agree), to maximize benefits.

## 6. Contributions and Study Implications

As discussed in the article, poor safety communication is a widespread and well-recognized problem in the construction industry [12,13,14,15]. The presented research advances existing knowledge by empirically evaluating workplace factors that can be managed and leveraged to foster safety communication among construction workers. The study makes several important contributions.

First, the study demonstrated that the established safety climate and the value assigned to workplace safety can influence whether safety information is exchanged efficiently at the crew level. The study results indicate that promoting a more positive safety climate can encourage workers to participate in providing and receiving safety information more actively. Accordingly, employers, contractors, and supervisors must devote attention to cultivating a positive safety climate to promote effective safety communication. For example, the management and the site leadership may (1) demonstrate their commitment and support to enhancing workplace safety, (2) craft and implement effective safety practices and policies at the workplace, and (3) reduce work-related pressure. 

Second, the study also showed that higher levels of cohesion can foster superior safety communication. Therefore, investing in efforts to promote crew-level cohesion can yield significant safety benefits. While past research has identified few factors that promote cohesion among group members (e.g., team-building activities, leader behaviors, personality of crew/team members) [42,43], there is a dearth of research on how cohesion can be improved particularly in the construction context. Therefore, although interventions such as team-building activities where unified goals are established (e.g., goal of achieving safety excellence) can be adopted in practice based on evidence from literature in other domains [63], future efforts may test these and newly proposed interventions particularly in the construction context. 

Third, the research suggests that a synergistic effect exists between safety climate and crew-level cohesion. Therefore, to promote effective safety communication, it is not sufficient to focus on only one of the two examined workplace factors. Workplaces that demonstrate a positive safety climate and includes crews that exhibit higher levels of cohesion will achieve superior levels of safety communication. The findings of the research will be useful to employers, contractors, and supervisors that desire to improve safety communication and safety performance.

Finally, because poor safety communication is a significant problem in the construction industry, employers, contractors, and supervisors may benefit from tracking safety climate and crew-level cohesion on a continual basis and intervene whenever necessary to foster effective safety communication among construction workers.

## 7. Study Limitations

Despite the study contributions, there are a few limitations of the study that must be acknowledged. First, all the variables examined in the current study including safety climate, crew-level cohesion, and safety communication were gathered during a single visit to the participating workplaces. Therefore, the research is cross-sectional in nature. While the evidence is sufficient to draw inferences with respect to associations, future efforts may focus on longitudinal research studies to draw stronger causal links between the examined variables. However, such an effort will require a significant amount of investment in terms of time and resources—particularly when the goal is to gather longitudinal data from over 50 projects. In addition, substantial commitment from the participating workplaces will be needed apart from the associated logistical challenges.

Second, the construction workplaces that participated in the current effort were all located in the Southeastern United States. Therefore there are limitations with the generalizability of the study findings to the general construction industry. Future research may replicate the effort while including a broader sample that includes workplaces from various geographical regions and nations.

Finally, while the current research effort quantified the exchange of safety information using a social network metric known as the network density, the effort did not particular capture the quality of such exchanges. Future efforts may complement the current effort by also considering the quality of the exchanged safety information.

## 8. Conclusions

Effective safety communication in construction workplaces is necessary to avoid hazard exposure and reduce injury likelihood [12,22]. However, previous research has demonstrated that systemic barriers to effective safety communication exist particularly in construction workplaces [12,13,14,15,27]. Unfortunately, these barriers can result in poor safety management and unexpected injuries [22]. Therefore, a better understanding of factors that impede and promote safety communication is necessary.

The presented research examined the role of safety climate and crew-level cohesion on safety communication levels. The research objectives were accomplished by gathering empirical data from 57 construction workplaces in the United States. More specifically, data relevant to the safety climate, the crew-level cohesion, and the safety communication levels were gathered using questionnaire survey instruments during a site visit.

The results of the research suggest that establishing a positive safety climate and promoting crew-level cohesion can foster superior safety communication levels among crew members. In addition, evidence of an interaction effect was found between safety climate and crew-level cohesion. More specifically, work crews that leverage the synergistic effects between safety climate and crew-level cohesion can expect even higher safety communication levels—than if they were to focus on either safety climate or cohesion individually.

The findings of this research can be leveraged to foster more effective safety communication among workers at the crew level. Apart from improving the communication and management of safety hazards, such efforts can translate to superior safety performance and fewer workplace injuries.

## Figures and Tables

**Table 1:** Survey instrument items to measure crew-level cohesion.

Crew-Level Cohesion Measurement Items
1. Our work crew often relies on each other to solve field-level problems
2. I feel comfortable to accept procedural suggestions from others in my crew
3. Crew members stick together outside the site or project
4. Every crew member tries to help each other if members have problems or challenges
5. I wish to work with the same crew in future projects
6. Our crew is united in trying to reach its goals for performance
7. We all take responsibility for any loss or poor performance by our crew as well as any achievements
8. Our crew members communicate freely about each of our personal responsibilities in getting this project done
9. Crew members are motivated to maintain unity among ourselves
10. There is a sense of team bonding amongst our crew members
11. I feel a sense of belonging to this crew
12. I am enthusiastic about being a member of this crew

**Table 2:** Survey instrument items to measure crew-level cohesion.

Variables	Mean	Std. Dev.	Zero-Order Correlations		
			1	2	3
1. Safety Climate Score	5.879	0.826	-	-	-
2. Crew-level Cohesion Score	5.986	0.785	0.716	-	-
3. Safety Communication Score	0.146	0.071	0.304	0.348	-

**Table 3:** Role of safety climate in fostering safety communication.

Predictors	Coefficient	Std. Error	*t*-Value	*p*-Value	LLCI	ULCI	*r* ^2^
Constant (*i*_1_)	−0.008	0.066	−0.115	0.909	−0.139	0.124	0.092
Safety Climate (*a*)	0.026	0.011	2.367	0.021	0.004	0.048	

Note: LLCI & ULCI = lower and upper limit confidence intervals; Significance level: *p*-value < 0.05.

**Table 4:** Role of crew-level cohesion in fostering safety communication.

Predictors	Coefficient	Std. Error	*t*-Value	*p*-Value	LLCI	ULCI	*r* ^2^
Constant (*i*^2^)	−0.042	0.069	−0.605	0.547	−0.180	0.097	0.121
Crew-level cohesion (*b*)	0.031	0.011	2.748	0.008	0.009	0.054	

Note: LLCI & ULCI = lower and upper limit confidence intervals; Significance level: *p*-value < 0.05.

**Table 5:** Synergistic effect between safety climate and crew-level cohesion.

Predictors	Coefficient	Std. Error	*t*-Value	*p*-Value	LLCI	ULCI	*r* ^2^
Constant	1.357	0.499	2.721	0.009	0.357	2.358	0.244
Safety Climate	−0.231	0.086	−2.702	0.009	−0.403	−0.060	
Crew-level cohesion	−0.228	0.089	−2.546	0.014	−0.407	−0.048	
Interaction	0.042	0.015	2.859	0.006	0.013	0.072	

**Table 6:** Conditional effects for various crew cohesion level.

Crew-Level Cohesion	Conditional Effect (*c* + *eC_crew_*)	Std. Error	*t*-Value	*p*-Value
4.735	−0.0313	0.0205	−1.5278	0.1325
5.037	−0.0183	0.0176	−1.036	0.3049
5.339	−0.0053	0.0156	−0.338	0.7367
5.641	0.0077	0.0147	0.5235	0.6028
5.943	0.0207	0.0152	1.3626	0.1788
6.245	0.0337	0.0169	1.9954	0.0512
6.2512	0.034	0.0169	2.0058	0.0500
6.396	0.0402	0.0181	2.2209	0.0307
6.698	0.0532	0.021	2.5282	0.0145
7.000	0.0662	0.0244	2.7061	0.0091
